# Chronic administration of mitochondrion-targeted peptide SS-31 prevents atherosclerotic development in ApoE knockout mice fed Western diet

**DOI:** 10.1371/journal.pone.0185688

**Published:** 2017-09-29

**Authors:** Meng Zhang, Hongting Zhao, Jing Cai, Huihui Li, Qi Wu, Tong Qiao, Kuanyu Li

**Affiliations:** 1 Department of Vascular Surgery, Drum Tower Clinical Medical College of Nanjing Medical University, Nanjing, P.R. China; 2 Jiangsu Key Laboratory for Molecular Medicine, Medical School of Nanjing University, Nanjing, China; 3 Department of Vascular Surgery, The Affiliated Drum Tower Hospital of Nanjing University Medical School, Nanjing, P.R. China; Beijing Key Laboratory of Diabetes Prevention and Research, CHINA

## Abstract

**Background:**

Oxidative stress and inflammatory factors are deeply involved in progression of atherosclerosis. Mitochondrion-targeted peptide SS-31, selectively targeting to mitochondrial inner membrane reacting with cardiolipin, has been reported to inhibit ROS generation and mitigate inflammation. The present study was designed to investigate whether SS-31 could suppress the development of atherosclerosis *in vivo*.

**Methods:**

Male ApoE^-/-^ mice (8 weeks old) fed with Western diet were treated with normal saline or SS-31 (1 mg/kg/d or 3 mg/kg/d) through subcutaneous injection for 12 weeks. Oil Red O staining was performed to evaluate area and sizes of the plaques. DHE staining and immunohistochemical staining of 8-OHDG was performed to assess the oxidative stress. The aorta ATP contents were assessed by the ATP bioluminescence assay kit. Immunohistochemical staining of CD68 and α-SMA and Masson’s trichrome staining were performed to evaluate the composition of atherosclerotic plaque. Biochemical assays were performed to determine the protein level and activity of superoxide dismutase (SOD). The levels of CD36, LOX-1 and ABCA1 were immunohistochemically and biochemically determined to evaluate the cholesterol transport in aorta and peritoneal macrophages. Inflammatory factors, including ICAM-1, MCP-1, IL-6 and CRP in serum, were detected through ELISA.

**Results:**

SS-31 administration reduced the area and sizes of western diet-induced atherosclerotic plaques and changed the composition of the plaques in ApoE^-/-^ mice. Oxidative stress was suppressed, as evidenced by the reduced DHE stain, down-regulated 8-OHDG expression, and increased SOD activity after chronic SS-31 administration. Moreover, systemic inflammation was ameliorated as seen by decreasing serum ICAM-1, MCP-1, and IL-6 levels. Most importantly, SS-31 administration inhibited cholesterol influx by down-regulating expression of CD36 and LOX-1 to prevent lipid accumulation to further suppress the foam cell formation and atherosclerotic progression.

**Conclusion:**

Administration of SS-31 prevents against atherosclerotic formation in ApoE^-/-^ mice suggesting that SS-31 might be considered to be a potential drug to prevent atherosclerotic progression.

## Introduction

Atherosclerosis, the main contributor to cardiovascular mortality, is a degenerative disease associated with oxidative stress and inflammatory factors [1]. The pathogenesis of atherosclerosis is complicated, involving multiple cell types including vascular endothelial cells, smooth muscle cells and pro-inflammatory cells such as macrophages [2]. Lipid accumulation within macrophages leading to foam cell formation and necrotic core growth accelerates atherosclerosis [3]. Limiting lipid retention and increasing lipid efflux in macrophages are promising strategy to prevent foam cell formation and atherosclerosis [4].

Oxidative stress probably due to the combination of highly reactive oxygen species (ROS) generation and impaired antioxidant defense is deeply involved in the pathogenesis of atherosclerosis [5]. ROS can modulate atherosclerosis progression partially by promoting DNA damage and accelerating cellular senescence. Meanwhile, mitochondrial DNA damage and dysfunction can augment ROS production, therefore, forming a positive feedback loop [6].

ROS can up-regulate the expression of oxidized low-density lipoprotein (ox-LDL) receptors on cell surface [7] including cluster of differentiation 36 (CD36) and lectin-like ox-LDL receptor-1 (LOX-1), which play important roles to take in ox-LDL [8]. Up-regulating CD36 expression promotes atherosclerosis in ApoE^-/-^ mice [9]. LOX-1 knockout mice exhibit reduced intima thickness, inflammation and atherosclerosis [10]. On the other hand, the ATP-binding cassette (ABC) transporters ABCA1 and ABCG1 remove accumulated cholesterol from macrophages onto extracellular receptors including high density lipoprotein (HDL) and apolipoprotein A-Ⅰ (apo A-Ⅰ) [8]. Interestingly, ABCA1 overexpression induces accumulation of proatherogenic lipoproteins and accelerates atherosclerosis [11]. Lack of ABCG1 has been reported to result in a mild augment of lesions in early stage of atherosclerosis, but causes retarded lesion progression in more advanced stage *in vivo* [12].

The notion of atherosclerosis as a chronic inflammatory disease has intensified research on the role of cytokines. Adhesion molecules, including intracellular adhesion molecule (ICAM), vascular adhesion molecule (VCAM) and monocyte chemoattractant protein (MCP), highly express in lipid-rich plaques thereby promoting recruitment of inflammatory cells (i.e., monocytes) into the plaque microenvironment leading to development of plaque lesion [13]. Considerable evidence suggests that impaired endogenous atheroprotective mechanisms occur at branch points in arteries, where the endothelial cells experience disturbed flow [14]. For example, absence of normal laminar shear stress reduces local production of endothelium-derived NO. In addition to inhibiting natural protective mechanisms, the disturbed flow can augment the production of certain leukocyte adhesion molecules (e.g. ICAM-1) [15]. Once adherent to the endothelium, the leukocytes penetrate into the intima. Research has identified candidate chemoattractant molecules responsible for this trans-migration, in which MCP-1 appears responsible for the direct migration of monocyte into the intima at sites of lesion formation [16, 17]. MCP-1 also contributes to the differentiation of the blood monocyte into the macrophage foam cells [18].

The peptide Szeto-Schiller (SS)-31 (D-Arg-dimethylTyr-Lys-Phe-NH_2_), belonging to a family of aromatic-cationic peptides, selectively targets to mitochondrial inner membrane reacting with cardiolipin [19], preventing ROS generation, improving ATP production, and decreasing oxidative stress [20]. These anti-oxidative effects have been shown to reduce ischemia-reperfusion injury [20], protect against neurodegeneration [21], and ameliorate insulin resistance caused by high-fat diet in several animal models [22]. Recently, we reported its inhibition of foam cell formation in RAW264.7 cells [23], which are a common cellular model of atherosclerosis. Here, we further investigate the *in vivo* effect of SS-31 on preventing the development of atherosclerosis in a mouse model.

## Materials and methods

### Animals

Forty-five male ApoE^-/-^ mice on C57BL/6 background (8-week-old) were obtained from the Model Animal Research Center of Nanjing University, Nanjing, China. ApoE^-/-^ mice were fed either a Western diet (0.2% cholesterol and 20% fat) in a pathogen-free animal facility with 12 h light and dark cycles under condition of controlled temperature (25°C). All experimental procedures and protocols were reviewed and approved by the Animal Investigation Ethic Committee of Nanjing University and were performed in accordance with the Guidelines for the Care and Use of Laboratory Animals from the National Institutes of Health, USA.

### Experimental protocols

SS-31 (synthesized by China Peptides Co, Ltd, Shanghai, China) or normal saline as a vehicle was administered to mice by subcutaneous injection for 12 weeks. Mice were randomly assigned to one of the following three groups (n = 15 each): Placebo (P, saline administration), SS-31 administration with dose of 1 mg/kg/d (M1) and 3 mg/kg/d (M3). The doses of SS-31 were chosen based on previous optimization in mouse models. The injection time was between 9 and 10 AM. No mice were dropped out during the experiments.

### Weight monitoring, blood and tissue collection, atherosclerotic plaque assessment

Body weight was recorded at the beginning and sacrifice day. After 12-week treatments, blood was collected under intraperitoneal pentobarbital anesthesia (40 mg/kg) via abdominal vena cava. Serum obtained was used to detect the lipid variables including total cholesterol (TC) and triglyceride (TG) by auto chemical analyzer (Beckman Coulter AU5421, CA). The rest serum was stored at -80°C for ELISA assays of ICAM-1, MCP-1, IL-6 and C-reactive protein (CRP) according to the manufacturer’s protocols (Elabscience Biotech Co, Ltd, Wuhan, China). Mice were then humanely euthanized by deep anesthesia with intraperitoneal pentobarbital anesthesia (80 mg/kg) followed by cervical dislocation and then organ removal. For analysis of lesion area, Oil Red O staining of area from aorta arch to abdominal aortic bifurcation were performed, whereas for analysis of atherosclerotic lesion size in aortic sinus, the proximal aorta attached to heart was harvested and fixed in 4% paraformaldehyde. Serial 6-μm-thick paraffin-embedded sections from the middle portion of the ventricle to the aortic arch were collected. The quantification of lesion area and size were performed using Image J software after Oil Red O staining by an experimenter who was blind to the treatment groups.

### Cell culture

Male 4- to 8-week-old ApoE^-/-^ (C57BL/6) mice weighing approximately 20–25 g were purchased from the Model Animal Research Center of Nanjing University. The mice were sacrificed by cervical amputation 4 days after an intraperitoneal injection of thioglycolate [24, 25]. The peritoneal cavity was washed with 10 mL cold PBS. Cells were harvested from the peritoneal cavity. Adherent macrophages were incubated for 18 hours with the RPMI-1640 medium containing 50 μg/ml human oxidized low-density lipoprotein (ox-LDL) in the presence/absence of SS-31 (50 nM or 100 nM). Oil Red O staining was performed to evaluate the foam cell formation. Cellular proteins were extracted and the levels of ABCA1, CD36 and LOX-1 were determined by western blotting analysis.

### Dihydroethidium staining, ATP measurement and superoxide dismutase activity detection

Fresh proximal aorta attached to heart were immediately embedded in Tissue-Tek OCT compound (Sakura Finetech Japan, Tokyo, Japan) and snap-frozen. Unfixed frozen samples were cut into 6-μm-thick sections and placed on glass slides. Dihydroethidium (DHE; 10 μM, Sigma-Aldrich) was applied to each tissue section, and the slides were subsequently incubated at 37°C in the dark for 30 min. Images were immediately obtained using a ZEISS HB050 inverted microscope system (Zeiss, Jena, Germany). Assessment of relative ATP contents in aorta was performed using the ATP bioluminescence assay kit (Beyotime, Haimen, China) following the manufacturer’s instruction. Aorta superoxide dismutase (SOD) activity was analyzed with a SOD assay kit (Nanjing Jiancheng Bioengineering Institute, Nanjing, China).

### Western blotting analysis

The sample (aorta or peritoneal macrophage) from each animal was harvested and homogenized in lysis buffer (Thermo Fisher Scientific, Waltham, UK), then placed on ice for 10 min. After centrifugation at 12,000 rpm for 10 min at 4°C, the supernatant was collected. Total proteins (35 μg/sample) were denatured at 95°C for 5 min in SDS and β-mercaptoethanol-containing sample buffer. The samples were subjected to electrophoresis on 10% or 12% SDS-polyacrylamide gels for 30 min at 80 V followed by 100 min at 100 V and then transferred onto nitrocellulose membrane (PALL, New York, NY) sheets for 90 min at 250 mA. After blocked with 5% fat-free milk for 90 min at room temperature, the blots were incubated at 4°C overnight with primary antibody SOD2 (1:1000 dilution, Abcam, Cambridge, MA), ABCA1 (1:500, Signalway Antibody LLC, College Park, MD), CD36(1:1000, Proteintech Group, Inc., Chicago, IN), LOX-1 (1:1000, Proteintech group, Inc.) or tubulin (1:2000, Sigma-Aldrich) as needed. Then the blots were incubated with HRP-conjugated secondary antibodies (goat anti-rabbit or mouse). Blotted-protein bands were visualized with enhanced chemiluminescence detection reagents (Thermo Fisher Scientific). Relative changes in protein expression were estimated from the mean pixel density using Image J, normalized to tubulin.

### Immunohistochemical staining and Masson’s Trichrome staining

Plaque composition was assessed in cross sections of aortic root by immunohistochemical staining for CD68 (macrophage marker) and α-SMA (smooth muscle cell marker), and Masson’s Trichrome staining for collagen. For immunohistochemical staining, after the endogenous peroxidase activity had been inhibited by hydrogen peroxide (H_2_O_2_) for 20 min, sections were incubated overnight at 4°C with primary antibodies 8-OHDG (1:200, Abcam), α-smooth muscle actin (α-SMA; 1:200, Abcam), CD68 (1:200, Abcam), CD36 (1:200, Proteintech Group), LOX-1 (1:200, Proteintech Group) and ABCA1 (1:200, Signalway Antibody LLC) as needs, followed by the appropriate secondary antibody (1:200, Santa Cruz Biotech). The sections were viewed under a light microscope (Zeiss). For quantitative analysis of images, five random fields were captured from different areas of a single section, and the intensity of positive staining was analyzed by Image J software and calculated as the percentage of total area of lesion or villa in each field by an experimenter who was blind to the treatment groups.

### Statistics analysis

The values were expressed as mean ± SEM. A one-way analysis of variance (ANOVA) followed by the Bonferroni test was performed using SPSS ver. 22.0 software (IBM Corporation, Armonk, NY, USA). Significance was considered at *p* < 0.05.

## Results

### Subcutaneous injection of SS-31 protects against atherosclerotic development in ApoE^-/-^ mice

**Fig 1:**
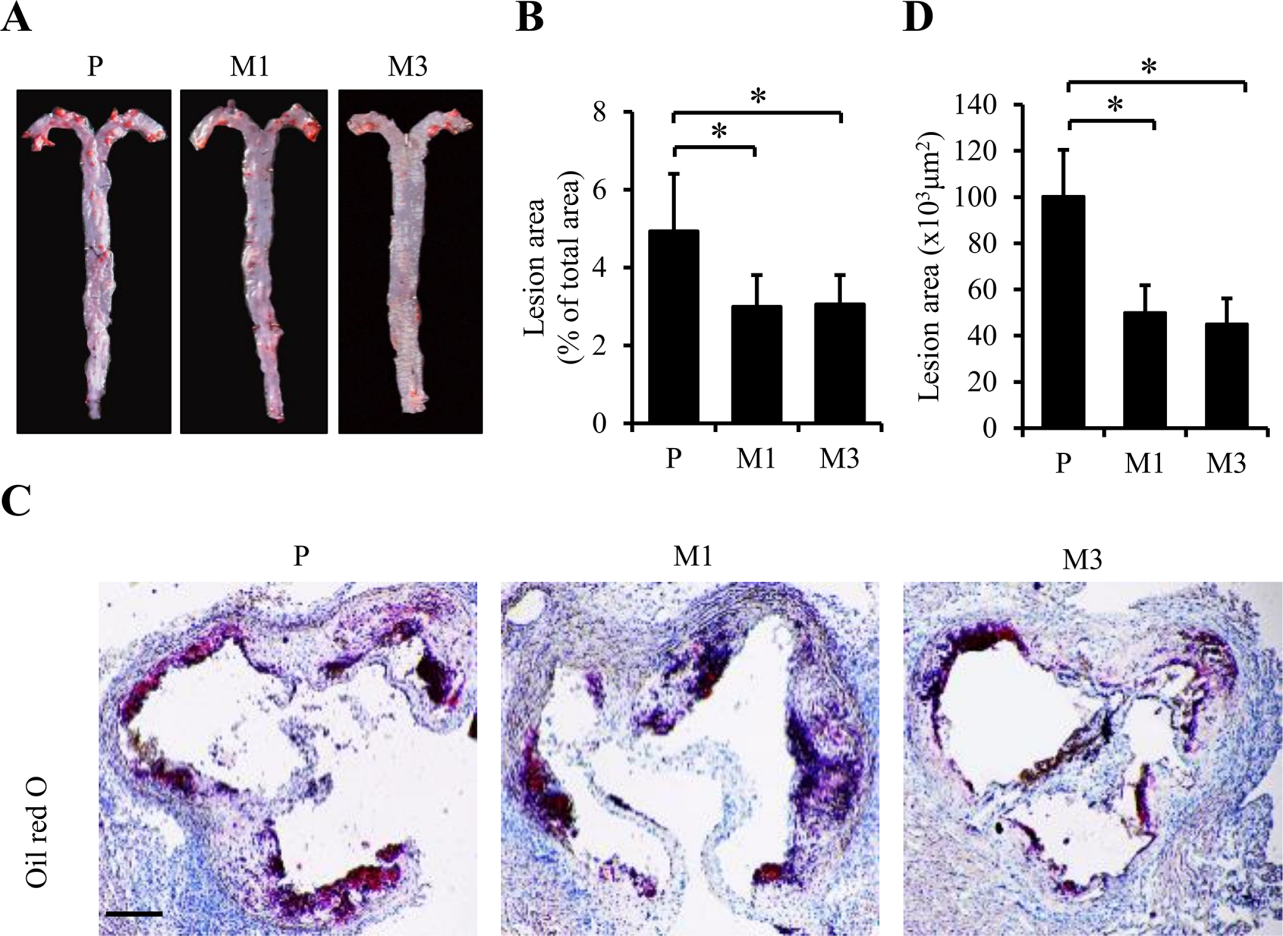
Chronic administration of SS-31 is associated with reduced atherosclerotic lesion area in ApoE^-/-^ mice. ApoE^-/-^ mice fed the Western diet were treated with either 1 mg/kg SS-31 (M1 group) or 3 mg/kg (M3 group) SS-31, or saline (P group) by subcutaneous injection daily for twelve weeks. Representative images of Oil Red O staining of aorta and aortic root are shown in (A) and (C). Quantification of positive area are shown in (B) for (A) and (D) for (C). Data represent the mean ± SEM. * *p* < 0.05. n = 5 for each group. The black scale bars represent 100 μm.

To explore the possible effect of SS-31 on atherosclerosis *in vivo*, ApoE^-/-^ mice fed the Western diet were treated with either 1 mg/kg SS-31 (M1 group) or 3 mg/kg (M3 group) SS-31, or saline (P group) by subcutaneous injection daily for twelve weeks. As shown in Table 1, body weight of the mice in each group was similar at both week 8 and 20 to each other. Plasma lipids including cholesterol and triglyceride were also similar among the three groups. Then, the atherosclerotic lesion formation was evaluated by En face Oil Red O staining of aorta. Oil Red O positive lesion area in M1 and M3 groups was significantly decreased compared to that in P group (2.99 ± 0.81% vs. 4.93 ± 1.47%, *p =* 0.033 for M1 *vs*. P; 3.05 ± 0.75% vs. 4.93 ± 1.47%, *p =* 0.035 for M3 *vs*. P in Fig 1A and 1B), indicating the reduced area and sizes of plaques after SS-31 administration in ApoE^-/-^ mice. Consistent with the reduction in the overall lesion area, decreased Oil Red O stained area in aortic roots was also observed in M1 and M3 groups compared to P group (6.51 ± 1.72% vs. 8.79 ± 1.77%, *p =* 0.045 for M1 *vs*. P; 6.29 ± 1.55% vs. 8.79 ± 1.77%, *p =* 0.046 for M3 *vs*. P in Fig 1C and 1D).

**Table 1:** Body weight and plasma lipids.

	Body weight (g)		
	P	M1	M3
Week 8	20.45±1.50	20.77±1.67	20.91±2.07
Week 20	26.34±1.32	26.59±1.71	26.85±1.64
Gain weight	5.89±1.16	5.81±1.35	5.94±2.46
**Plasma lipids**			
Cholesterol (mmol/L)	16.38±0.38	16.50±0.21	16.62±0.13
Triglycerides (mmol/L)	0.99±0.32	1.06±0.37	1.11±0.40

Plasma lipids include cholesterol and triglyceride. Data represent the mean ± SEM.

* *p* < 0.05., n = 8 for each group. No significant difference was found.

**Fig 2:**
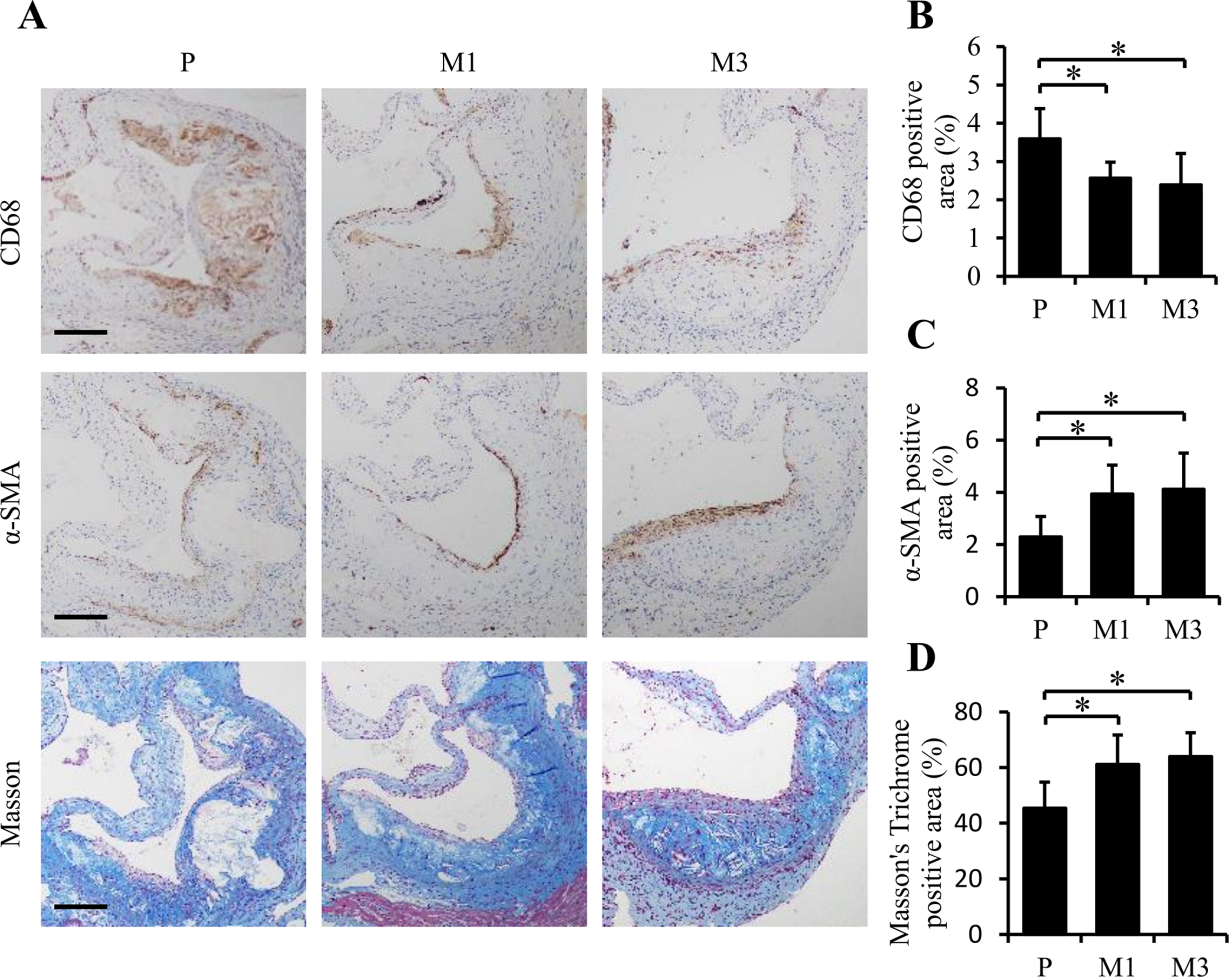
SS-31 administration modulates the composition of atherosclerotic plaques in ApoE^-/-^ mice. Representative images of immunohistochemical staining are shown in (A) for CD68 (as a marker of macrophages), α-SMA (as a marker of smooth muscle), and Masson staining for collagenous fibers in aorta root. Quantification of stained area as a percentage of lesion area is given in (B), (C), and (D), respectively. Data represent the mean ± SEM. * *p* < 0.05. n = 5 for each group. The black scale bars represent 50 μm.

To further investigate the plaques, the aortic roots of mice in each group were immunohistochemically stained to assess the composition, such as macrophages, smooth muscle, and collagen by the levels of CD68 and α-SMA (encoded by ACTA2) expression and the stained intensity of Masson’s Trichrome, respectively. In accordance with the decrease in overall lesion area in M1 and M3 groups compared to P group, a significant decrease of CD68 immuno-stained macrophages was observed (2.56 ± 0.42% *vs*. 3.58 ± 0.79%, *p* = 0.033 for M1 *vs*. P; 2.39 ± 0.82% vs. 3.58 ± 0.79%, *p =* 0.046 for M3 *vs*. P in Fig 2A and 2B). Interestingly, significant increases were revealed for α-SMA immuno-stained area on the surfaces of the plaques (3.93 ± 1.11% *vs*. 2.29 ± 0.79%, *p =* 0.034 for M1 *vs*. P; 4.11 ± 1.39% *vs*. 2.29 ± 0.79%, *p =* 0.033 for M3 *vs*. P in Fig 2A and 2C) and collagen-stained areas (61.13 ± 10.61% *vs*. 45.41 ± 9.4%, *p =* 0.019 for M1 *vs*. P; 63.9 ± 8.65% *vs*. 45.41 ± 9.4%, *p =* 0.011 for M3 *vs*. P in Fig 2A and 2D). These results suggest that the decreased lesion area of mice in M1 and M3 groups is associated with less advanced, more stable plaques than that of mice in P group.

### Subcutaneous injection of SS-31 suppresses oxidative stress and improved ATP synthesis in the aorta of ApoE^-/-^ mice

**Fig 3:**
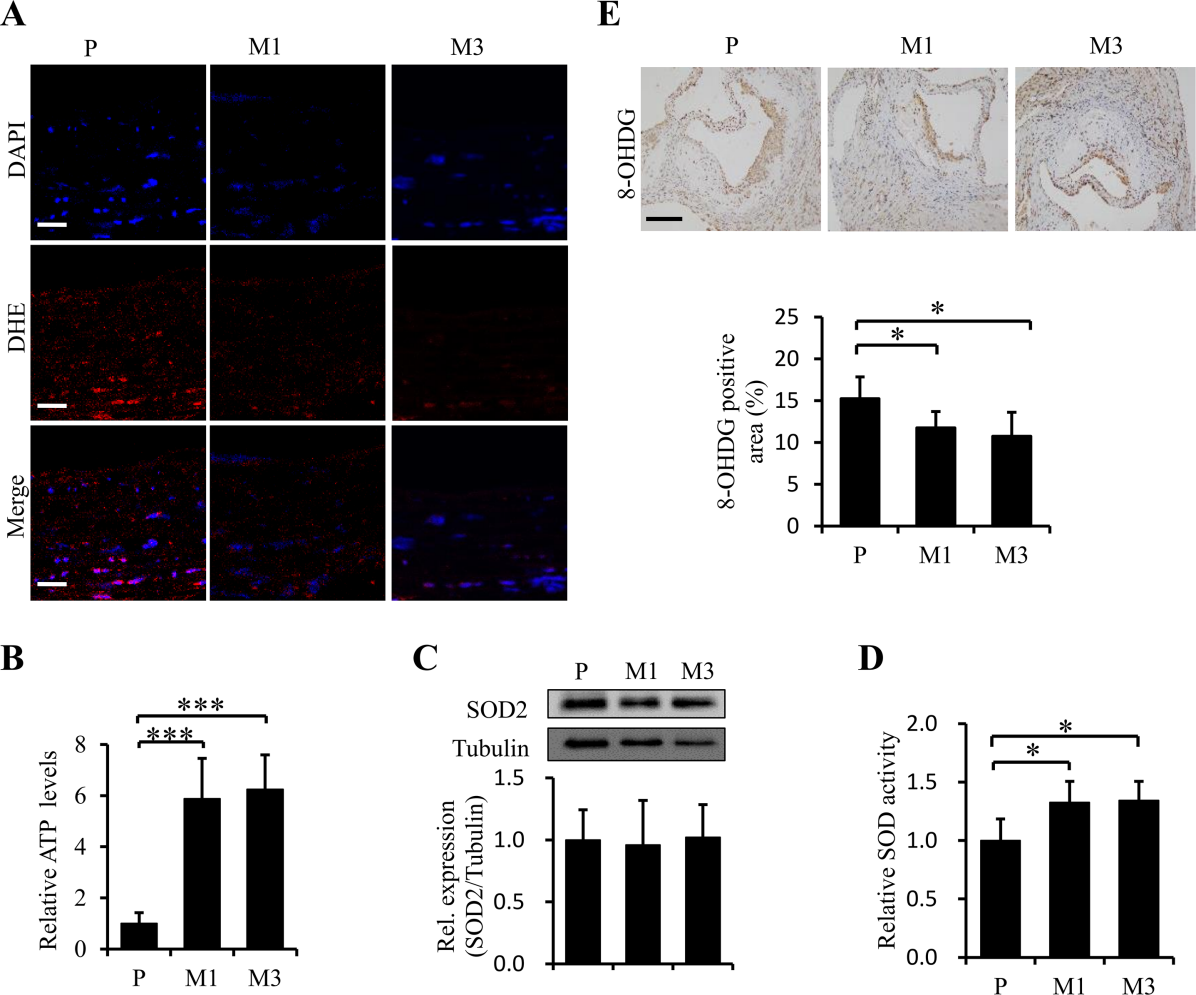
Chronic administration of SS-31 improves ATP production, reduces ROS accumulation and increases the competence against oxidative stress and damage in aorta. Representative images of DHE stain are shown in (A). ATP production (B) in aorta was measured immediately after the samples were prepared in 5 mice from each group. Western blotting analysis of SOD2 expression in aorta is shown in (C) from 4 independent experiments. Enzymatic activity of SOD (n = 5 for each group) is shown in (D). Representative images of immunohistochemical staining for 8-OHDG and quantification of stained area as a percentage of lesion area are presented in E (n = 5 for each group). Data represent the mean ± SEM. * *p* < 0.05, ***p < 0.001. The white scale bars represent 20 μm. The black scale bars represent 50 μm.

Oxidative stress is deeply involved in the pathogenesis of atherosclerosis[5] and SS-31 is considered as an efficient mitochondrion-targeted antioxidant, so we examined whether SS-31 could reduce ROS accumulation and improve mitochondrial function in the aorta of ApoE^-/-^ mice after twelve-week administration. The levels of ROS and cellular ATP were evaluated with DHE fluorescence staining and with a biochemical kit, respectively. The results revealed that SS-31 administration reduced the cellular level of ROS in the vascular wall (Fig 3A) and improved energetics of aorta (*p <* 0.001, Fig 3B). Then the cellular ROS scavenging ability was assessed by determining the protein level and enzymatic activity of SOD. The protein level of SOD2 in aorta was kept constant (*p >* 0.05, Fig 3C), but the SOD activity in aorta was significantly up-regulated in mice treated with SS-31 (*p =* 0.044 for M1 *vs*. P; *p =* 0.033 for M3 *vs*. P, Fig 3D) compared with that in mice treated with saline. We further analyzed the protective effect of SS-31 against the oxidative damage of DNA by ROS with 8-OHDG immunohistochemical staining. The results showed a significant reduction of the 8-OHDG-positive area of aortic root in mice treated with SS-31 (11.74 ± 1.96% *vs*. 15.24 ± 2.60%, *p* = 0.043 for M1 *vs*. P; 10.75 ± 2.85% *vs*. 15.24 ± 2.6%, *p =* 0.031 for M3 *vs*. P, Fig 3E) compared to that in mice treated with saline. Collectively, our results indicate that SS-31 provides protective effects against atherosclerotic development most likely through reducing oxidative stress to improve energetics of aorta.

### Subcutaneous injection of SS-31 ameliorates systemic inflammation in ApoE^-/-^ mice

**Fig 4:**
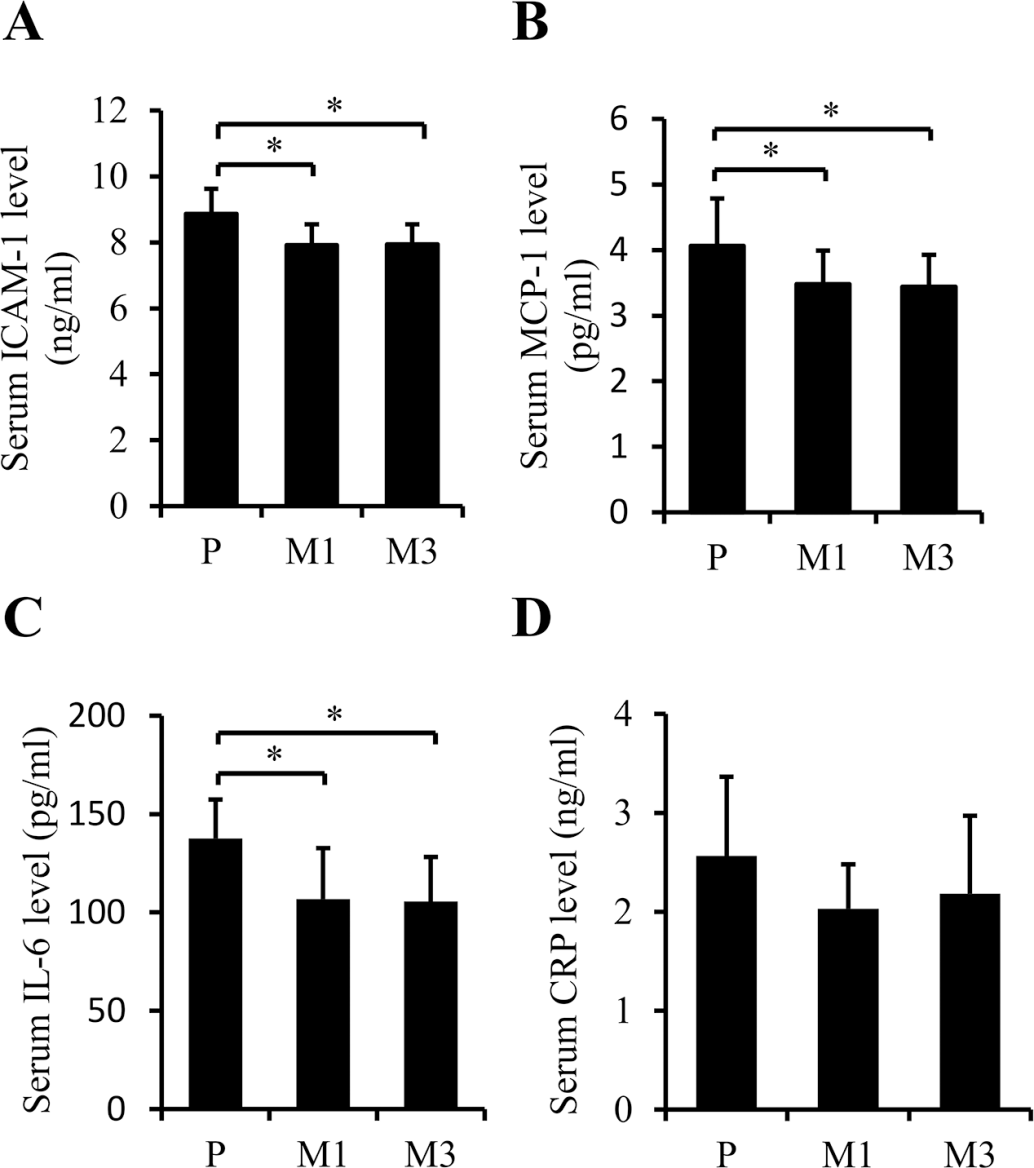
Subcutaneous injection of SS-31 ameliorates systemic inflammation in ApoE^-/-^ mice. (A) Serum ICAM-1. (B) Serum MCP-1. (C) Serum IL-6. (D) Serum CRP. n = 10 for P group, n = 10 for M1 group, n = 6 for M3 group. Data were collected with ELISA assays and represent the mean ± SEM. * *p* < 0.05.

Chronic inflammation is one of the pathogenic features of atherosclerosis [13]. ICAM-1 and MCP-1 are the major chemokines involved in accelerating the adhesion of monocytes/macrophages onto endothelium and subsequent transmigration into intima [16, 26]. In consistence with the reduction of macrophages in plaques, ICAM-1 and MCP-1 were significantly decreased in serum of mice treated with SS-31 (*p =* 0.012 and *p =* 0.030 for M1 and M3 *vs*. P, respectively (comparison order same in the following figures), in Fig 4A; *p =* 0.026 and *p =* 0.046; Fig 4B). Macrophages within the vessel wall could release pro-inflammatory cytokines including IL-6, IL-1β, and tumor necrosis factor (TNF)-α, which will further mediate distant inflammatory effects, such as activating hepatic genes encoding acute phase reactant fibrinogen, C-reactive protein (CRP) and serum amyloid A[27]. The levels of CRP and IL-6 were determined and found to be dropped in serum of mice treated with SS-31 significantly for IL-6 (*p =* 0.044; *p =* 0.027; Fig 4C), not for CRP (*p =* 0.133; *p =* 0.369; Fig 4D). Taken together, the results suggest that daily injection of SS-31 could overall ameliorate systemic inflammation in ApoE^-/-^ mice.

### Subcutaneous injection of SS-31 modulates the lipid uptake of macrophages in the aorta of ApoE^-/-^ mice

**Fig 5:**
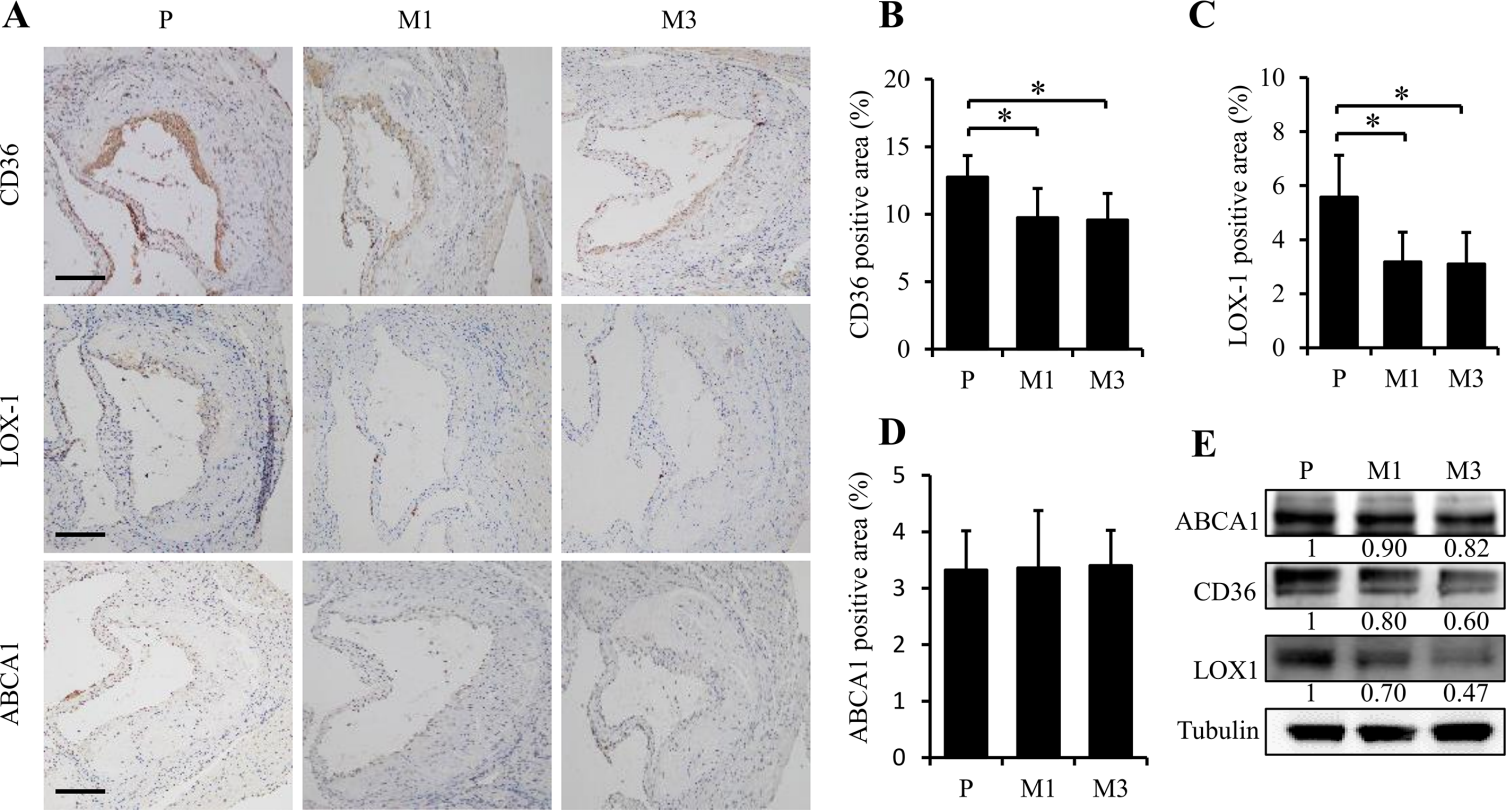
Administration of SS-31 modulates the lipid uptake of macrophages in the aorta of ApoE^-/-^ mice. Representative images of immunohistochemical staining for CD36, LOX-1, and ABCA1 in aortic root are presented in (A). The black scale bars represent 50 μm. Quantification of stained area as a percentage of lesion area is given in (B) for CD36, (C) for LOX-1, and (D) for ABCA1. Western blotting analysis of ABCA1, LOX-1, and CD36 expression in aorta is shown in (E). Data represent the mean ± SEM. * *p* < 0.05. n = 5 for each group.

Foam cell formation is a critical event of early atherosclerotic plaque. Uncontrolled uptake of oxidized low-density lipoprotein (ox-LDL), excessive cholesterol esterification and impaired cholesterol release contribute to accumulation of cholesterol ester (CE) stored as lipid droplets and subsequently trigger the formation of foam cells. Therefore, we used immunohistochemical staining to detect the expression of CD36 and LOX-1, two principal receptors responsible for ox-LDL influx, and ABCA1, one of the important transporter mediating cholesterol efflux. As shown in Fig 5, CD36 (M1 *vs*. P, *p =* 0.038 and M3 *vs*. P, *p =* 0.024 in Fig 5B) and LOX-1 (M1 *vs*. P, *p =* 0.022 and M3 *vs*. P, *p =* 0.021 in Fig 5C) were significant down-regulated by SS-31 treatment. However, no difference was observed for the expression of ABCA1 (*p >* 0.05, Fig 5D) after SS-31 administration. The western blotting data also supported the above observation (Fig 5E).

**Fig 6:**
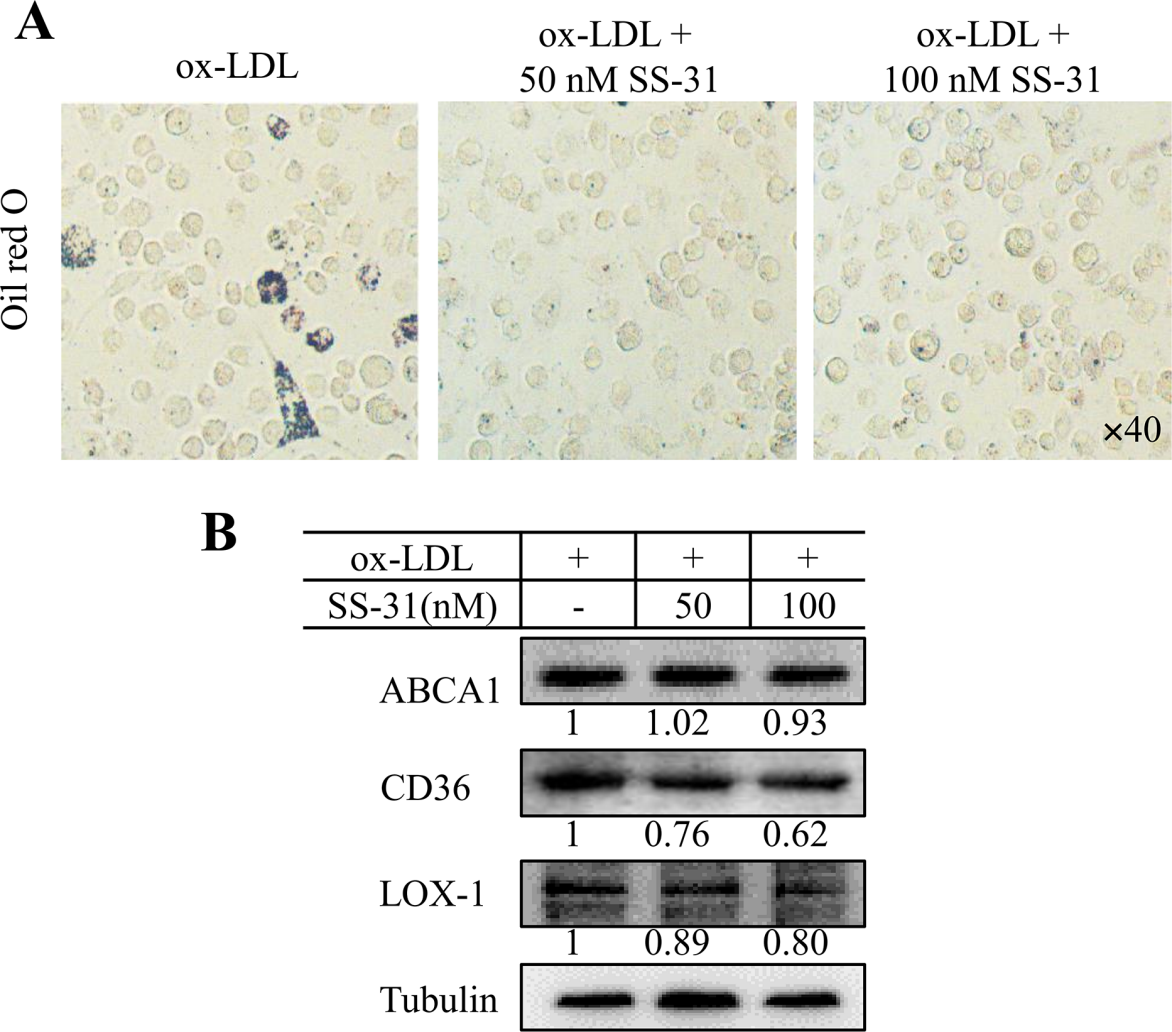
SS-31 treatment reduced ox-LDL-induced lipid accumulation in peritoneal derived macrophages, associated with decreased lipid influx. Peritoneal derived macrophages were exposed to ox-LDL (50 μg/mL) in the presence or absence of SS-31 (50 or 100 nM) for 24 h. Samples were stained for lipid detection or collected for biochemical assays. (A) Oil Red O-stained peritoneal derived macrophages (x40). The dark color indicates the lipids and/or lipoproteins. (B) The expression levels of LOX1, CD36, and ABCA1 detected by western blot.

We further performed the *in vitro* study by using peritoneal derived macrophages to evaluate the effect of SS-31 on lipid influx. As shown in Fig 6A, SS-31 reduced ox-LDL-induced lipid accumulation in peritoneal derived macrophages. Lipid flux-related protein levels of LOX1, CD36, and ABCA1 were detected by western blot. The results showed that CD36 and LOX1 expression levels decreased after SS-31 treatment, and ABCA1 kept constant, suggesting that SS-31 treatment down-regulates cholesterol influx, but does not affect cholesterol efflux. Taken the *in vivo* and *in vitro* data together, it is demonstrated that SS-31 treatment inhibits the lipid accumulation, which is associated with decrease of CD36 and LOX-1 expression to suppress ox-LDL uptake, further prevents foam cell formation.

## Discussion

Atherosclerosis is a chronic inflammatory disease initiated by arterial endothelium injury and promoted by uptake of ox-LDL into macrophages, transforming them into foam cells [1]. Lipid lowering drugs, such as statins, are the main means to prevent atherosclerosis progression in current clinical practices [28]. Here we show that SS-31 could ameliorate Western diet-induced deterioration of atherosclerosis in ApoE^-/-^ mice. Histological data revealed decreased macrophage and increased α-SMA-positive cell population, and increased collagen content after SS-31 treatment, presenting changed plaque composition to a more stable phenotype. These features are associated with the decreased lesion area and size in SS-31-treated groups, implying less advanced and vulnerable plaques after SS-31 administration. Pathologically, not only did SS-31 reduce ROS level, decrease oxidative damage, and improve ATP production, but also inhibited inflammatory response. More interestingly, we found that SS-31 treatment decreased the cholesterol accumulation through inhibition of lipid uptake in the plaque area of aorta. These findings suggest that SS-31 could be considered as a therapeutic drug for atherosclerosis.

It has been proved that SS-31 can prevent ROS generation, improve ATP production and decrease oxidative stress in many cell/animal models of human diseases, including ischemia-reperfusion injury [20], neurodegeneration [21], insulin resistance [22], heart failure [29] and hypertensive cardiomyopathy [30]. In consistence with this, our cell model of atherosclerosis has demonstrated that SS-31 treatment inhibits foam cell formation in RAW264.7 cells [23]. In current *in vivo* study, daily injection with SS-31 ameliorated atherosclerotic development in ApoE^-/-^ mice. In general, along with the progress of atherosclerosis, the number of α-SMA positive smooth muscle cells increased [1, 6]. Because smooth muscle cells make up the smooth muscle cap, which helps to stabilize the plaque in the late phase [2], increased ɑ-SMA stained areas indicate less advanced and more stable of the plaques after SS-31 administration (Fig 2). This preclinical effects are in accordance with therapeutic prospects for atherosclerosis. On the one hand, a pursuing approach is to change the clinical course by reducing the size and composition of plaques. On the other hand, it is also important to stable the “vulnerable plaques” which typically have a large lipid core, thin fibrous cap, and inflammatory cell infiltration. SS-31 is effective in both aspects as shown in this study.

Oxidative stress plays an important role in the pathogenesis and development of atherosclerosis [5]. Our data from the vasculature of ApoE^-/-^ mice injected with SS-31 demonstrated decreased superoxide levels by DHE fluorescence staining compared to the controls (Fig 3A). SOD, glutathione peroxidase, and catalase can convert ROS into water and balanced molecules [31]. Although the protein level of SOD2 in aorta was not changed, we found significantly increased SOD activity in aorta of SS-31 injection mice (Fig 3C and 3D). We assumed that the mechanism by which SS-31 reduces ROS levels in aorta could be, at least partially, through the ROS scavenger with SOD activity. Excessive ROS may damage the vascular smooth muscle cells and endothelial cells and promote atherosclerosis progression closely associated with DNA damage and cellular senescence [6]. The role of mitochondrial DNA damage and impaired mitochondrial function in atherosclerosis has been highlighted recently and studies have even suggested that atherosclerosis is a mitochondrial disease [32]. We showed that DNA damage represented by positive area of 8-OHDG immunohistochemistry in aortic root was significantly down-regulated by SS-31 treatment. Mitochondria are the powerhouses of the cell, responsible for generating ATP that levels of aorta were remarkably increased by daily injection with SS-31 in our study. Taken together, SS-31 treatment thoroughly improves the quality of mitochondria probably by increasing the ability of defense against oxidative stress and protecting the mitochondria from damage.

Inflammation and hypercholesterolemia are two key etiologic factors of atherosclerosis [1, 33]. The initial pace of atherosclerosis is an injury to the arterial endothelium. Then the injured endothelium can secrete adhesion molecules and chemokines to recruit leukocytes into the intima [1]. Persistent inflammation increases expression of macrophage scavenger receptor and increases the uptake of ox-LDL to form foam cells [1]. Foam cells secrete more inflammatory factors and further promote migration of leukocytes and smooth muscle cells into the lesion area [1]. Therefore, anti-inflammation intervention is considered as a promising way to treat atherosclerosis [34]. Recently, in a model that reproduced *in vivo* interaction between leukocytes and endothelial cells, anti-TNF treatment diminished leukocyte-endothelial interaction induced by inflammatory stimuli [35]. Clinical trials in patients with rheumatoid arthritis have shown that tocilizumab blocking both membrane-bound and circulating IL-6 receptor increases HDL levels and improves endothelial function and decreases aortic stiffness [36]. Consistent with decreased macrophages in lesion, daily injection with SS-31 significantly decreased the serum level of several chemokines and adhesion molecules including MCP-1, ICAM-1 and IL-6. However, Western diet-induced hypercholesterolemia was not changed by SS-31 treatment (Table 1), supporting the notion that hypercholesterolemia is a principal risk factor and leads to an inflammatory response within the microvasculature, whereas anti-inflammation intervention does not appear to correlate with reduction in low-density lipoprotein levels in spite of, to some extent, improvement of atherosclerosis.

Limiting inflammation did not lower the systematic cholesterol, but significantly instigate the lipid metabolism of the local plaques. The imbalance between uptake of ox-LDL and cholesterol release triggers the formation of foam cells [8], which is a critical event of early atherosclerotic plaque. In this study, we found that SS-31 could down-regulate the expressions of CD36 and LOX-1, which play an important role to take in ox-LDL, in aorta suggesting the blockage of ox-LDL uptake. ABCA1 plays a critical role in mediating the active transport of intracellular cholesterol and phospholipids to apoA-Ⅰ, the major lipoprotein in HDL [8]. Here we found that the expression of ABCA1 in aorta did not change after three month’s injection of SS-31, suggesting that the anti-atherosclerotic effect of SS-31 on the reduction of foam cell formation is mainly attributed to the inhibition of cholesterol influx rather than efflux.

In summary, our study indicates that SS-31 reduces oxidative stress, circulation inflammation, and cholesterol influx in the plaques to prevent Western diet-induced atherosclerotic progessive development in ApoE^-/-^ mice. From the mechanism how SS-31 works [19, 37], we think that the primary effect of SS-31 is via protecting mitochondrial integrity and quality to prevent mitochondrion-dependent ROS generation. Our findings raise the possibility of SS-31 for treatment of atherosclerosis.
